# The associations of human genetic variations with airway microbiome, environmental exposures, and respiratory health

**DOI:** 10.1128/msystems.00442-26

**Published:** 2026-07-29

**Authors:** Shifen Xu, Lei Yang, Jingyuan Gao, Yunfeng Shi, Xin Tang, Hanqin Cai, Liling Yang, Yuanyuan Han, Lifeng Lin, Ruilin Meng, Jiufeng Sun, Wei-jie Guan, Tao Tang, Wensheng Shu, Chao Cao, Xue-yan Zheng, Zhang Wang, Xinzhu Yi

**Affiliations:** 1 Guangdong Engineering Research Center of Precision Detection and Modulation of Human Microbiome, School of Life Sciences, South China Normal University 12451, Guangzhou, Guangdong, China; 2 Guangdong Provincial Center for Disease Control and Prevention, Guangzhou, Guangdong, China; 3 State Key Laboratory of Respiratory Disease, National Clinical Research Center for Respiratory Disease, Guangzhou Institute for Respiratory Health, The First Affiliated Hospital of Guangzhou Medical University, Guangzhou, Guangdong, China; 4 Department of Thoracic Surgery, Guangzhou Institute for Respiratory Health, The First Affiliated Hospital of Guangzhou Medical University, Guangzhou, Guangdong, China; 5 Pulmonary and Critical Care Medicine Department, Huizhou Central People’s Hospital, Huizhou, Guangdong, China; 6 Department of Respiratory and Critical Medicine, Key Laboratory of Respiratory Disease of Ningbo, The First Affiliated Hospital of Ningbo University 117881, Ningbo, Zhejiang, China; Qingdao University, Qingdao, Shandong, China

**Keywords:** respiratory microbiome, air pollution, host genetic variation, Mendelian randomization

## Abstract

The intricate interactions between environmental exposures, the respiratory microbiome, and host genetic variations remain inadequately understood in the context of respiratory health. This study utilized sputum metagenomic data from 1,651 individuals in our previous cohort to elucidate these associations. Mendelian randomization indicated that air pollutants (e.g., SO_2_, CO, and PM_2.5_) were associated with lung function, which is potentially mediated by microbes, such as *Actinomyces*, *Haemophilus influenzae*, and *Veillonella* spp. Several genetic loci associated with respiratory microbiome variation were found to be linked to genotype-dependent associations between environmental exposures and lung function. For bacteria, the *MEOX1* locus (rs1973191819) was associated with lower *Filifactor alocis* abundance under air pollutant exposure. The *FAM110D* (1:26157175) and *USP36* (rs1343834070) loci showed associations with higher levels of certain pathogenic taxa (e.g., *Ralstonia pickettii*, *Neisseria*) and lower levels of the commensal *Oribacterium*, increasing chronic obstructive pulmonary disease (COPD) risk. For fungi, *DNAJC18* and *CCDC57* loci exhibited associations with *Candida* and *Penicillium* abundance, respectively. These genotype-dependent associations between the microbiome and environmental exposures provide insights into airway dysbiosis and susceptibility to respiratory diseases.

**IMPORTANCE:**

This study reveals why individuals exposed to identical air pollution exhibit varying degrees of respiratory severity, pointing to a critical missing link: our genetics. While pollution is a known disease trigger, our findings demonstrate that host genetic variation actively regulates and shapes the respiratory microbiome under environmental stress. By mapping specific genetic loci to pollutant-driven bacterial shifts, this work elucidates how host genetics filters environmental risks to govern microbial homeostasis. These results underscore the necessity of incorporating host-microbiome genetic regulation into environmental health research. Ultimately, this study shifts the paradigm toward personalized medicine, enabling the early identification of at-risk individuals and the development of targeted, microbiome-informed interventions.

## INTRODUCTION

Sustained exposure to environmental pollutants impairs lung function and increases the risk of chronic respiratory diseases, such as chronic obstructive pulmonary disease (COPD), primarily through oxidative stress and inflammatory pathways (1–3). The respiratory microbiota plays a pivotal role in maintaining epithelial barrier integrity and immune homeostasis, with its dysbiosis closely linked to airway inflammation and diminished lung function (4–9). Additionally, numerous studies have emphasized the influence of human genetics on microbial composition, identifying multiple genomic loci associated with microbial traits. Genome-wide association studies (GWAS) also indicate that lung function measures, such as the ratio of forced expiratory volume in one second to forced vital capacity (FEV_1_/FVC), exhibit substantial heritability (10, 11), suggesting that host genetic variants may modulate individual susceptibility to environmental particulate matter or cigarette smoke (12). Despite these insights, a systematic investigation into how human genetics shape the interplay among respiratory microbiota, environmental exposures, and lung function is still lacking, hindering our understanding of the molecular mechanisms underlying respiratory health and disease.

The heritability of specific bacterial taxa and host genetic loci associated with microbial abundance has been well documented in the human gut (13–16). However, for the respiratory tract, the heritability of the microbiota and the contribution of specific host genetic loci in shaping airway microbial composition remain poorly understood (10, 17, 18). Recent advancements in two-step Mendelian randomization (MR) provide a powerful framework to dissect potential mediation pathways linking exposures, intermediate traits, and disease outcomes (19). For instance, a large MR study demonstrated links among smoking, lung function, and COPD in the context of COVID-19 (20). However, the mediation of the respiratory microbiome on environmental exposures and respiratory health within the context of host genetic background remains unclear. Elucidating these interactions is essential for advancing our understanding of the host-microbiome-environment network underlying respiratory diseases.

As the respiratory tract is directly connected to the external environment, the respiratory microbiota, serving as a gatekeeper at the host-environment interface (21), is continuously exposed to a wide range of environmental factors. Given that an imbalanced respiratory microbiota has been strongly associated with increased airway inflammation, decreased lung function, and exacerbations of asthma and COPD (9, 22–24), it may act as a potential mediator of the detrimental effects of environmental exposures on lung function. Although several studies have demonstrated that environmental exposures can alter the stability and diversity of the respiratory microbiota, potentially mediating their adverse effects on respiratory health (25–27), the underlying mechanisms remain largely unexplored.

In this study, metagenome-GWAS (mgGWAS) was performed using sputum samples from 1,651 individuals in our previous cohort. Specifically, the genetic associations between the airway microbiome, air pollution, and lung function were systematically evaluated through GWAS. Subsequently, a two-step MR framework was employed to validate the mediating role of the microbiome and its genetic regulation. Additionally, heritable core taxa in the respiratory tract and their corresponding host loci were identified through a fine-mapping approach. Genotype-stratified association analyses were conducted using generalized linear models (GLMs) to elucidate how host genetic variants and microbial factors jointly influence the effects of environmental exposures on lung function. This study aims to unravel the host genetic-environment-microbiome-lung function regulatory network, offering novel insights and potential targets for precision prevention and intervention in chronic respiratory diseases.

## RESULTS

### Profiling host genetic variants from airway metagenomic data

**Fig 1:**
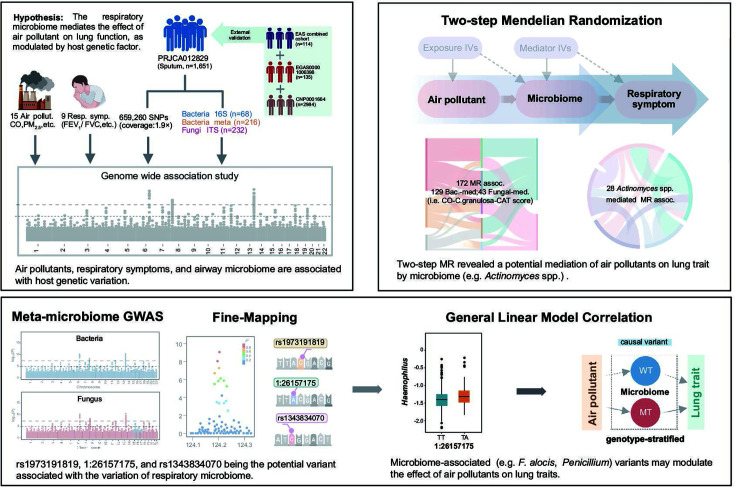
Study design illustrating host genetic-dependent microbiome modulation of air pollution effects on respiratory phenotypes.

The metagenomic sequencing data yielded an average of 2.13 × 10^7^ high-quality reads per sample. Of these, an average of 93.6% of the reads were derived from the human host, providing approximately 1.9× coverage of the human genome (a low-coverage genotyping regime; see Limitations), and were subsequently subjected to SNP calling (Fig. 1). After comprehensive quality filtering, 659,260 high-confidence SNPs were retained from 1,450 samples (Fig. S1A). The distribution of SNPs across the 22 autosomes showed no strong regional preferences, though a high density of SNPs was observed on chromosome 19. Principal component analysis of the SNP data revealed no obvious population stratification among the analyzed samples. The distribution of genetic variants across functional regions aligned with expected patterns, with no statistically significant deviations (Fig. S1B).

To assess data quality, the missing rate and genotype composition were evaluated at the sample level (Fig. S1C). The vast majority of samples exhibited a low missing rate (<2%), and their genotype distributions (homozygous reference, heterozygous, and homozygous variant) were consistent with expectations, indicating no obvious outliers. At the SNP level (Fig. S1D), the minor allele frequencies (MAFs) showed a comparable distribution across all autosomal chromosomes, while Hardy–Weinberg equilibrium (HWE *P* ≥ 0.05) tests revealed that most variants conformed to population expectations, with only a small fraction significantly deviating. Furthermore, the distribution of observed versus expected heterozygosity was centered near zero, suggesting no systematic bias (Fig. S1D). These results support the overall reliability of the SNP data set after quality control.

### Host genetic variations are associated with environmental exposures, respiratory traits, and microbiome features

Genome-wide association analyses of 15 air pollutants identified 247 SNPs (suggestive significance at *P* < 1.0 × 10^–5^) significantly associated with environmental exposures (Table S1), providing genetic insights into host responses to environmental challenges. KEGG enrichment analysis indicated that genes mapped from exposure-associated loci were enriched in pathways related to glycosaminoglycan biosynthesis (heparan sulfate/heparin) (*P* < 0.001), FcγR-mediated phagocytosis (*P* = 0.002), and the endocytosis pathway (*P* = 0.002) (Table S2; Fig. S2A). These pathways are involved in core biological processes, such as antimicrobial defense, anti-inflammatory regulation, and immune responses (28, 29).

Across the GWAS of nine respiratory traits, 909 SNPs (suggestive significance at *P* < 1.0 × 10^–5^) were identified, providing insights into the genetic basis of pulmonary function (Table S1). These SNPs were significantly enriched in KEGG pathways, including the AMPK signaling pathway (*P* = 0.006), bacterial invasion of epithelial cells (*P* = 0.008), and endocytosis (*P* = 0.012) (Table S2; Fig. S2B). In addition, several identified loci map to genes that have been previously implicated in pulmonary phenotypes (Table S3). For instance, rs933939908 (*P* = 8.9 × 10^–6^) associated with the CAT score was mapped to *WWOX*, a gene previously implicated in multiple independent GWAS of various lung-related traits, such as lung function, COPD, asthma, and COVID-19 (10, 30, 31). Two novel loci significantly associated with FEV_1_/FVC were identified: rs34712586 (*P* = 6.1 × 10^–6^) in *TNFRSF8* and 16:11130010 (*P* = 2.4 × 10^–6^) in *CLEC16A*. Both genes have been reported in previous studies to harbor loci associated with multiple respiratory traits, including asthma, lung function, and respiratory diseases, across several independent cohorts (32, 33) (Table S3). Within *CLEC16A*, several suggestive GWAS loci have been implicated in asthma in multiple GWAS. A novel locus at 1:204614191, located in *LRRN2*, was identified and has previously been associated with genetic variations linked to COPD. Additionally, the locus at 2:111056668, located in *ACOXL*, was significantly associated with pre-COPD (*P* = 5.9 × 10^–6^) and has also been implicated in genetic studies of asthma (34) (Table S3).

### Replication in independent cohorts validates host genetic associations with the respiratory microbiome

Replication analyses using independent metagenomic data sets from multiple East Asian cohorts (PRJCA007879, PRJNA714488, PRJNA858501, and PRJNA991321,) and previously reported mgGWAS summary statistics (CNP0001664 from the oral microbiome and EGAS00001006398 from our previous study [35]) confirmed the robustness of these associations. A major strength of this study is the use of induced sputum, which provides a more direct window into the lower airway microbiome compared with nasal or oral swabs (Table S13). Combined with a large sample size (*n* = 1,651) and detailed host genetic and environmental exposure profiling (Tables S2 to S4), our study offers valuable insights into the genetic regulation of the lower respiratory microbiome and its role in mediating environmental effects on lung function.

For East Asian cohorts, microbial taxonomic profiles and GWAS analyses were re-performed to ensure consistency. Cross-dataset comparisons revealed substantial consistency, with our mgGWAS data set sharing 50,991, 40,426, and 88,797 common SNPs and overlapping with 86, 72, and 82 microbial species across the three reference data sets (Fig. S3). Several microbial species common to both the discovery and replication cohorts exhibited consistent associations with host genetic variation (Fig. S3), and their overlapping genes were similarly enriched in the PI3K–Akt signaling pathway (KEGG) and the small GTPase-mediated signaling pathway (Gene Ontology [GO]) (Table S2; Fig. S2C through E). Furthermore, several SNPs associated with microbial taxa (*P* < 1 × 10^–4^) were robustly replicated across cohorts. For instance, rs59426107 and 7:100102207, which are associated with *Haemophilus influenzae*, were consistently identified in East Asian cohorts (Fig. S2F; Table S4).

### Potential microbial mediation of associations between environmental exposures and respiratory traits

**Fig 2:**
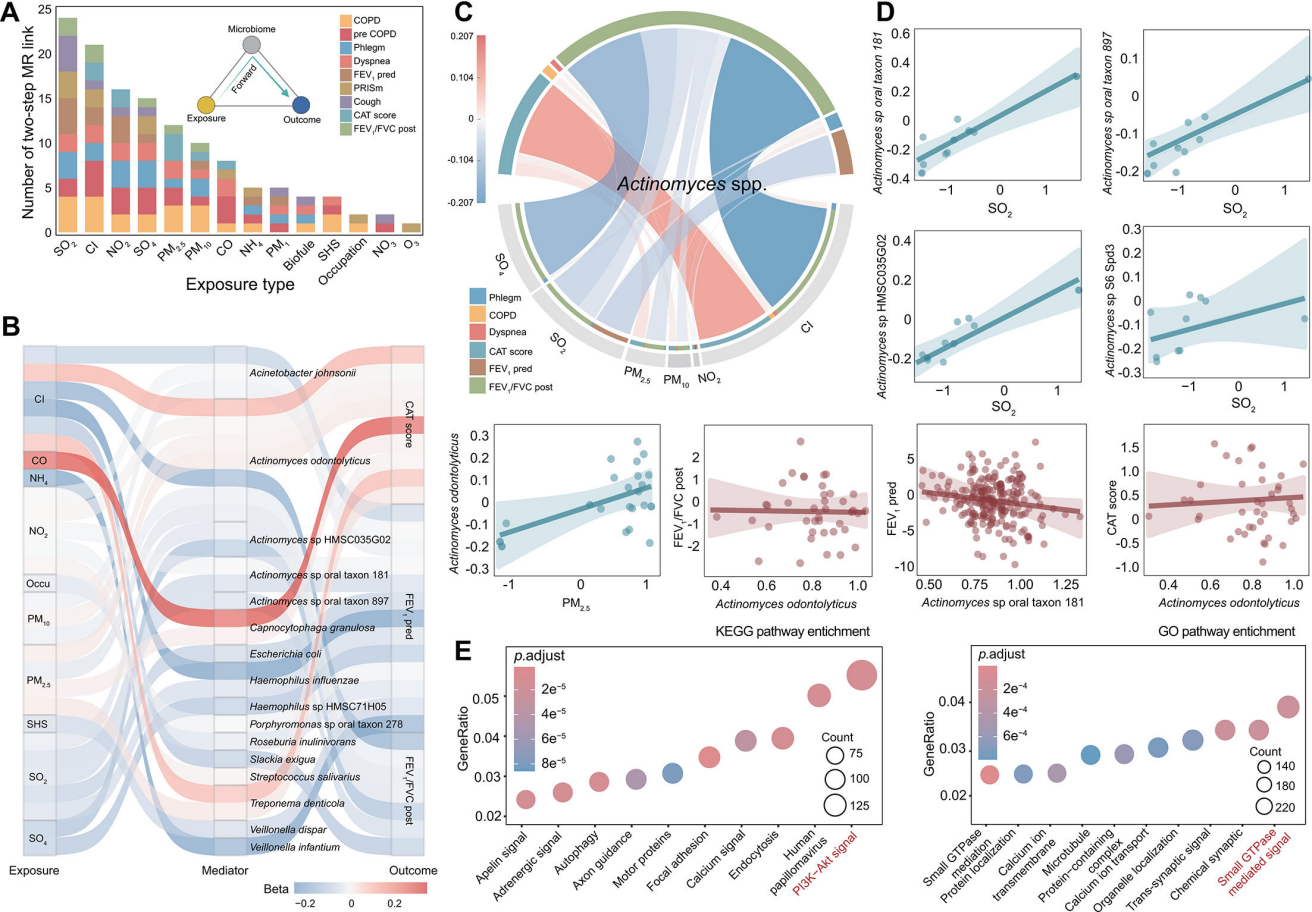
Mendelian randomization and pathway enrichment analyses of bacteria-mediated effects of environmental exposures on health outcomes. (**A**) Bar plot showing the number of bacterial-mediated MR associations between air pollutant exposures and health outcomes. The *x*-axis represents specific air pollutant types, and the *y*-axis indicates the count of MR associations for each pollutant. Different colors correspond to distinct health outcomes. (**B**) Sankey diagram illustrating the detailed bacterium-mediated effects of exposures on three health outcomes. The left, middle, and right panels represent exposures, bacterial taxa, and pulmonary function indices, respectively. The color gradient encodes the magnitude of the mediation effect (β_*m*_). (**C**) Chord diagram displaying the mediating role of multiple *Actinomyces* species linking various exposures to different health outcomes. Exposures are arranged along the lower semicircle, while health outcomes occupy the upper semicircle, with colors indicating distinct outcomes. The chord color represents the magnitude of the mediation effect (β_*m*_). (**D**) Scatter plot of SNP-specific associations for *Actinomyces* species. The *x*-axis represents SNP effects on exposure, and the *y*-axis represents SNP effects on the outcome. Each dot corresponds to a single genetic variant, and the fitted line denotes the causal estimate from MR analysis. Blue dots indicate the first MR step (exposure → *Actinomyces*), and red dots indicate the second MR step (*Actinomyces* → health outcome). (**E**) Bubble plot summarizing KEGG and GO pathway enrichment for genes mapped from genome-wide significant loci (*P* < 1 × 10^–5^) associated with bacterial taxa exhibiting mediating effects. The *x*-axis lists pathway names, and the *y*-axis shows the gene ratio (the proportion of enriched genes). Bubble size reflects the number of enriched genes, while color indicates the adjusted significance level.

A two-step MR analysis was performed to investigate potential mediation using cross-sectional data (observational correlations at a single time point), with 15 air pollutants as environmental exposures, 216 bacterial taxa with relative abundance as mediators, and 9 respiratory traits as outcomes. A total of 129 associations reached significance between 14 environmental exposures, 39 bacterial taxa, and 9 respiratory traits (Table S5). Among these, SO_2_, Cl, and NO_2_ were the top pollutants that harbored the greatest number of bacteria-mediated associations (Fig. 2A). SO_2_ exposure showed associations with eight pulmonary traits that were potentially mediated by the largest number of bacterial species, including *Actinomyces*, *Haemophilus influenzae*, and *Neisseria perflava*. *Capnocytophaga granulosa* exhibited the largest potential mediating (β_*m*_ = 0.38) effect on the association between CO exposure (β_1_ = 1.754, inverse-variance weighted [IVW] *P*_adj_ = 0.046) and CAT scores (β_2_ = 0.214, IVW *P*_adj_ < 0.001). This was followed by *H. influenzae*, which showed a potential mediating role in the association between increased Cl exposure and reduced FEV_1_ pred (β_*m*_ = –0.27). *Veillonella infantium* showed a potential mediating effect linking higher NH_4_ levels to decreased FEV_1_ pred (β_*m*_ = –0.24) (Table S5; Fig. 2B). In addition, the genus *Actinomyces* was associated with the greatest number of exposures-outcome relationships (*n* = 28), driven largely by *Actinomyces odontolyticus* (*n* = 19) (Fig. 2C and D). Pathway enrichment analysis of significant genes from these bacterial mediators. The PI3K–Akt signaling pathway (KEGG) and small GTPase-mediated signaling (GO) were most significantly enriched, both playing critical roles in lung diseases (Fig. 2E; Table S6) (36, 37).

A substantial proportion of these mediation effects were observed in COPD (24 associations) and pre-COPD (22 associations) (Table S5). COPD was significantly associated with multiple exposures, including biofuel, Cl, NO_2_, NO_3_, PM_2.5_, PM_10_, second-hand smoking, SO_2_, and SO_4_, which were potentially mediated by bacterial taxa, such as *A. odontolyticus*, *Eubacterium brachy*, *H. influenzae*, *Tannerella forsythia*, and *Veillonella dispar*. In contrast, pre-COPD was primarily linked to 10 exposures (including Cl, CO, PM_1_, PM_10_, PM_2.5_, SO_2_, and SO_4_), mediated by 13 bacterial species, including *Capnocytophaga granulosa*, *Capnocytophaga leadbetteri*, *Filifactor alocis*, *Streptococcus salivarius*, *Treponema denticola*, and *Veillonella parvula* (Table S5; Fig. S4A).

Additionally, 43 fungus-mediated associations involving 9 exposures, 15 fungal genera, and 9 lung function outcomes were identified (Table S5). Among these, NO_2_ exhibited the highest number of fungi-mediated associations (*n* = 11) (Fig. S4B). The largest potential mediating effect was observed for *Sarocladium* (β_*m*_ = –1.34) in the association between increased CO and reduced FEV_1_/FVC post (post-bronchodilator FEV_1_/FVC), followed by *Geosmithia* (β_*m*_ = –0.92) and *Bipolaris* (β_*m*_ = –0.72) (Table S5; Fig. S4C). NH_4_ showed a significant positive association with the COPD outcome, a relationship potentially mediated by the fungus *Coprinopsis* (β_*m*_ = 0.058). Significant genes derived from fungi mediators were notably enriched in the endocytosis pathway (*P* < 0.001) and small GTPase-mediated signal transduction (*P* < 0.001) (Table S5; Fig. S4D), both of which are involved in host immune responses to microbes (38–40).

### Microbiome-associated loci were identified in meta-GWAS

A total of 225 bacterial traits demonstrated sufficient SNP-based heritability and were retained for the meta-microbiome GWAS analysis. This set included 107 continuous traits (58 species-level, 49 genus-level) (Table S7) and 118 binary traits (109 species-level, 9 genus-level) (see Materials and Methods; Table S8). *Sphingomonas* (heritability *h*^2^ = 0.941, *P* < 0.001; genomic inflation factor λ = 0.937) and *Prevotella pallens* (*h*^2^ = 0.989, *P* < 0.001; λ = 1.088) exhibited the highest heritability estimates among all quantitative traits analyzed, indicating a substantial influence of host genetics on their abundance (Table S7). These point estimates warrant caution; for low-prevalence taxa like *Sphingomonas* (35.1% prevalence), restricted maximum likelihood (REML) estimates can be upwardly inflated. Additionally, these values may be further biased by inherent compositional data constraints and microbial co-abundance networks (14, 41).

**Fig 3:**
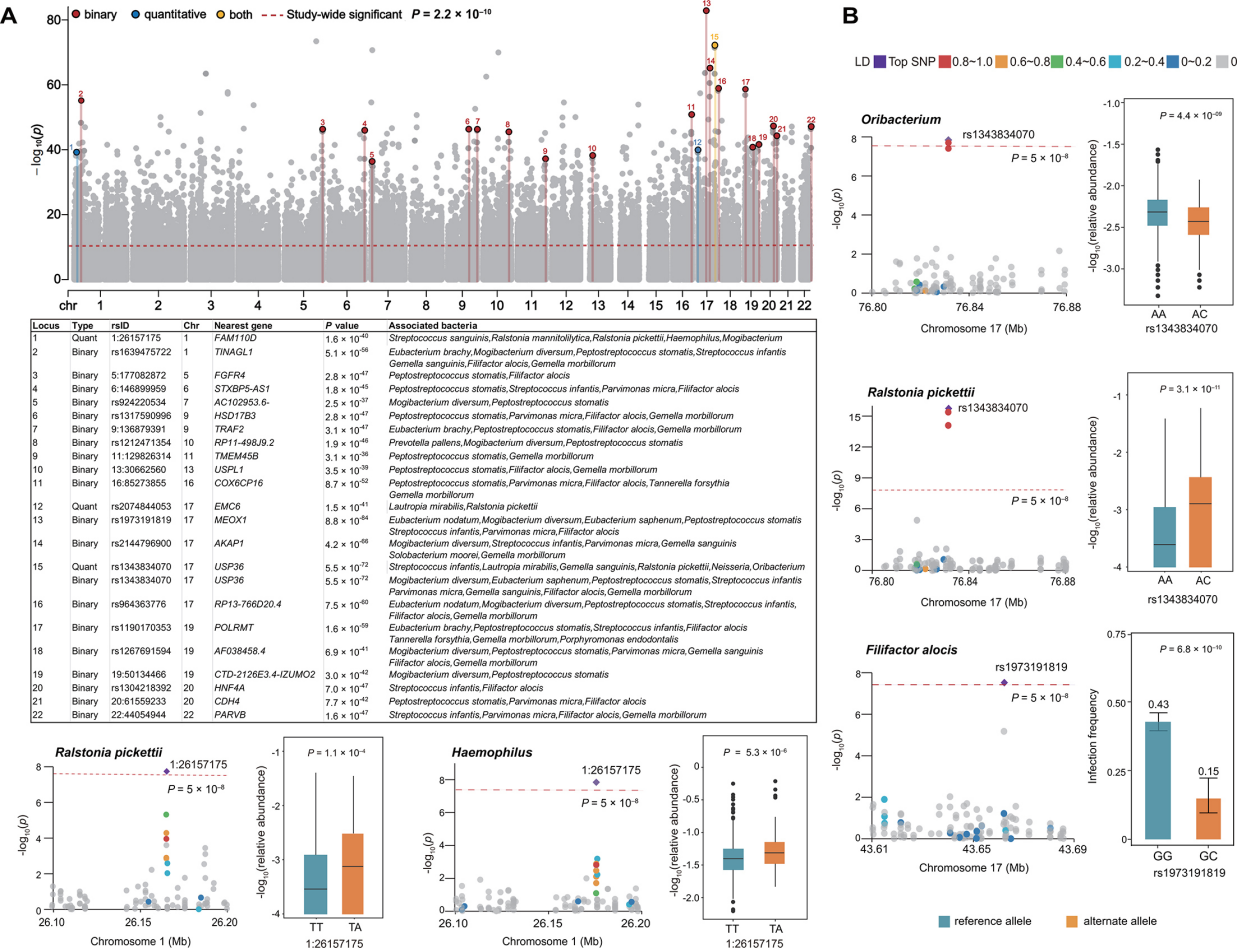
Host genetic associations with bacterial traits revealed by meta-GWAS and locus-specific analyses. (**A**) Manhattan plot of the bacterial meta-GWAS results. The red dashed line indicates the study-wide significance threshold (*P* < 2.2 × 10^–10^). Highlighted loci (±1 Mb around lead SNPs) are color-coded: red for loci significant in at least two binary bacterial traits, blue for at least two quantitative traits, and yellow for both. The accompanying table summarizes 22 loci, including locus number, bacterial trait type, SNP ID, chromosome, annotated gene, meta-GWAS *P* value, and associated bacterial taxa (*P* < 5 × 10^–8^). (**B**) Regional association plot of the most significant bacterial species and genus with the three causal loci. The red dashed line marks the GWAS significance threshold (*P* < 5 × 10^–8^), and point color represents linkage disequilibrium (LD) strength with the causal variant. Boxplots display bacterial relative abundances (log_10_-transformed) across wild-type and mutant genotypes at loci 1:26157175 and rs1343834070. *P* values (Wilcoxon test) are shown above each plot. The bar chart illustrates bacterial infection rates across rs1973191819 genotypes, with proportions labeled above bars and significance determined by Fisher’s exact test.

Study-wide significance thresholds for microbiome traits were established at *P* < 2.2 × 10^–10^ (5 × 10^–8^/225), identifying 25,839 significantly bacterial-associated SNPs. This study identified 20 distinct genetic loci associated with the presence/absence of bacterial taxa and 3 loci associated with bacterial abundance, with at least two bacterial taxa represented in each locus (Fig. 3A). The variant rs1973191819, located upstream of the *MEOX1* gene (Table S9), exhibited the strongest study-wide significant association (*P* = 8.77 × 10^–84^) with the presence/absence of seven bacterial taxa, all of which reached genome-wide significance (*P* < 5 × 10^–8^). These seven bacterial taxa included *Eubacterium nodatum* (*h*^2^ = 0.468 for presence/absence, λ = 1.190, Table S8), *Mogibacterium diversum* (*h*^2^ = 0.808, λ = 0.944), *Eubacterium saphenum* (*h*^2^ = 0.622, λ = 1.221), *Peptostreptococcus stomatis* (*h*^2^ = 0.903, λ = 0.978), *Streptococcus infantis* (*h*^2^ = 0.785, λ = 1.233), *Parvimonas micra* (*h*^2^ = 0.734, λ = 1.103), and *Filifactor alocis* (*h*^2^ = 0.955, λ = 1.043). The second variant, rs1343834070 (*P* = 5.54 × 10^–72^), located downstream of the *USP36* gene, showed study-wide significant associations with both quantitative and binary traits (Fig. 3A; Table S9). It was significantly associated with the abundance of six bacterial taxa, including *S. infantis* (*h*^2^ = 0.660 for abundance, λ = 0.97, Table S7), *Lautropia mirabilis* (*h*^2^ = 0.162, λ = 1.197), *Gemella sanguinis* (*h*^2^ = 0.944, λ = 1.082), *Ralstonia pickettii* (*h*^2^ = 0.367, λ = 1.436), *Neisseria* (*h*^2^ = 0.382, λ = 1.54), and *Oribacterium* (*h*^2^ = 0.553, λ = 1.18).

Among these loci, the strongest significant variant, rs1973191819, was identified as a candidate variant associating the presence/absence of seven bacterial taxa (Fig. 3B; Fig. S5). Individuals in the GC cohort were significantly associated with a lower infection frequency of *F. alocis* (freq_GC_ = 0.15, freq_GG_ = 0.43, Fisher test: *P* < 0.001) (Fig. 3B), as well as *E. nodatum*, *M. diversum*, *E. saphenum*, *P. stomatis*, *S. infantis*, and *P. micra* (Fig. S5A). Two loci (1:26157175 and rs1343834070) were identified as candidate variants associated with the abundance of multiple bacterial taxa (Fig. 3B; Fig. S5). Variant 1:26157175, located upstream of *FAM110D*, was linked to increased abundances of *R. pickettii*, *Haemophilus*, *Streptococcus sanguinis, and Ralstonia mannitolilytica*, as well as decreased abundance of *Mogibacterium* in the TA cohort relative to the TT cohort (Fig. 3B; Fig. S5B). Variant rs1343834070 was associated with reduced levels of *R. pickettii*, *Oribacterium*, *S. infantis*, and *G. sanguinis*, but higher levels of *L. mirabilis* and *Neisseria* in the AC cohort compared to the AA cohort (Fig. 3B; Fig. S5C). Sensitivity analyses using different covariate adjustment models (including age + sex, age + sex + BMI, and models with/without genetic principal components) showed highly consistent effect sizes, with changes in ranging from 0.001 to 0.028 (all <6%), and confidence intervals for the lead SNPs associated with *G. sanguinis*, *R. pickettii*, and *S. infantis* (Fig. S5D). These results indicate that the identified host genetic associations with airway microbial taxa are robust to covariate adjustment. Notably, this genetic association showed high taxon-specificity, with robust cis-eQTL evidence for *USP36* that is consistently strong in lung tissues across both bacterial contexts, whereas the downstream GWAS association with microbiota abundance is highly significant for *R. pickettii* (*P* = 3.92 × 10^–16^), but completely absent for *Staphylococcus aureus* (*P* = 0.883) (Fig. S6).

**Fig 4:**
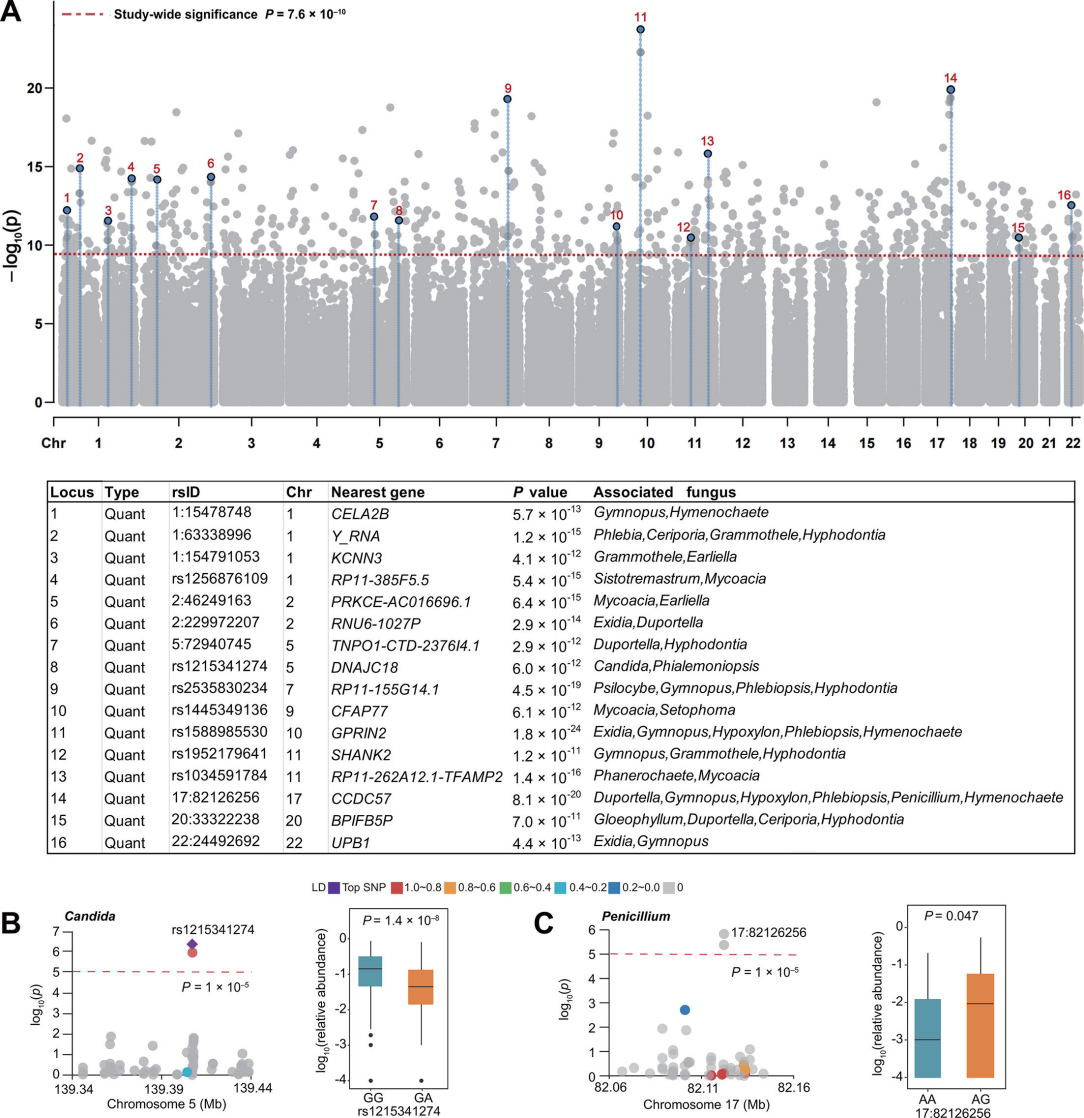
Integrative analysis of fungal GWAS and fungus-mediated effects of environmental exposures on health outcomes. (**A**) Manhattan plot of the fungal meta-GWAS results. The red dashed line denotes the study-wide significance threshold (*P* < 7.6 × 10^–10^). Highlighted loci (±1 Mb around lead SNPs) are color-coded for loci significant in at least two quantitative traits. The accompanying table summarizes 16 loci, including locus number, bacterial trait type, SNP ID, chromosome, annotated gene, meta-GWAS *P* value, and associated fungal genus (*P* < 1 × 10^–5^). (**B**) Regional association plot of *Candida* and *Penicillium* with loci rs1215341274 and 17:82126252. The red dashed line marks the GWAS significance threshold (*P* < 1 × 10^–5^), and point color represents LD strength with the causal variant. (**C**) Boxplots display bacterial relative abundances (log_10_-transformed) across wild-type and mutant genotypes at two loci. *P* values (Wilcoxon test) are shown above each plot.

A total of 66 fungal genus-level traits exhibited significant SNP-based heritability and were included in the meta-microbiome GWAS, comprising 37 quantitative and 29 binary traits (Tables S7 and S8). This study identified 843 SNPs that reached study-wide significance (*P* < 7.6 × 10^–10^) across 258 candidate loci. The strongest association signal for fungal abundance was identified at locus 10:46560118 (*P* = 1.77 × 10^–24^), which is located upstream of the *GPRIN2* gene (Fig. 4A; Table S10). Following this, high-resolution fine-mapping of the lead SNPs (*n* = 16) was conducted across the 258 candidate loci. This analysis revealed rs1215341274, mapped to *DNAJC18*, as a candidate variant showing a statistically significant association with *Candida* abundance (*P* = 6 × 10^–12^) in the airway mycobiome. Moreover, *Candida* abundance significantly differed between wild-type and mutant-type cohorts at the rs1215341274 locus (Fig. 4B). Additionally, another locus, 17:82126256, mapped to *CCDC57*, was identified, which was slightly associated with the abundance of *Penicillium* (*P* = 1.08 × 10^−6^) (Fig. 4B).

### Genotype-dependent microbial features mediate the association between environmental exposure and respiratory traits

To assess the modifying effect of these variants on relationships between environmental exposures and respiratory traits, this study performed genotype-stratified association analyses using GLM, comparing wild-type and mutant-type subpopulations defined by three microbiome-related variants (rs1973191819, 1:26157175, and rs1343834070). Four environmental exposures (CO, SO_2_, SO_4_, and smoking) were consistently associated with a decreased FEV_1_/FVC post ratio. Notably, the decline rate of FEV_1_/FVC post was more pronounced in the mutant-type cohort compared to the wild-type cohort (Table S11). Genotype-stratified associations in this section are reported at a suggestive threshold (*P*_adj_mutant_ < 0.1, *P*_adj_wild_ > 0.1) and are intended to identify variant-modified candidate pathways.

**Fig 5:**
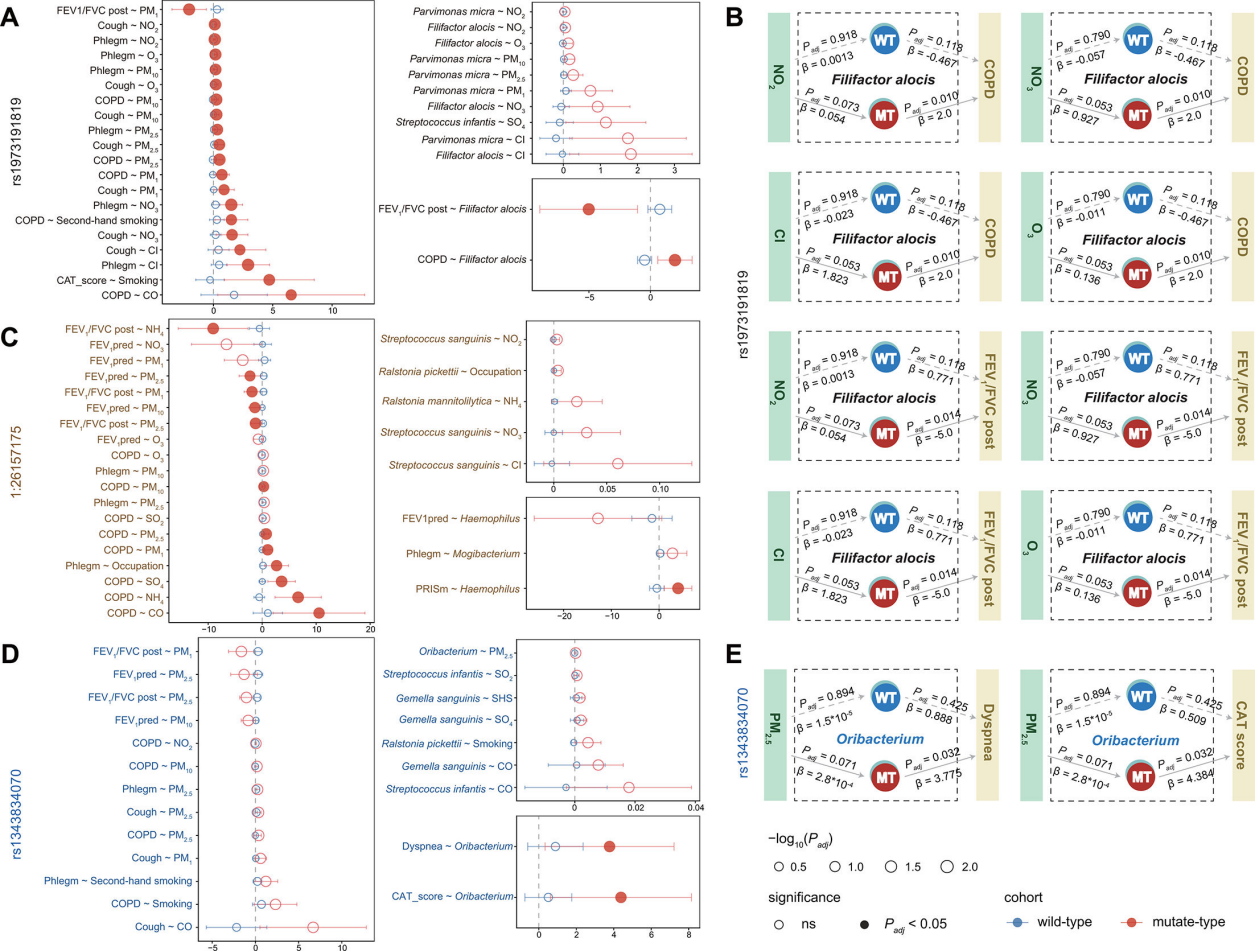
Potential microbiome-mediated associations between environmental exposures and health outcomes, stratified by host genotype. (**A, C, D**) Bubble plots showing associations between exposures, microbiome features, and health outcomes stratified by genotype at three causal loci (rs1973191819, 1:26157175, rs1343834070). The *x*-axis represents effect sizes, and the *y*-axis indicates associated variables. Blue and red circles represent wild-type and mutant groups, respectively. Circle size corresponds to –log_10_ (*P*_adj_), and error bars represent 95% confidence intervals. Solid circles denote confirmatory associations (*P*_adj_ < 0.05), and dashed circles denote exploratory associations (*P*_adj_ < 0.1). (**B and E**) Mediated models established by genotype-stratified associations. For rs1973191819, associations between exposures (Cl, O_3_, NO_2_, NO_3_) and health outcomes (FEV_1_/FVC post-bronchodilator and COPD) mediated by *Filifactor alocis* are presented. For rs1343834070, associations between PM_2.5_ and CAT score or dyspnea mediated by *Oribacterium* are shown. Arrows indicate estimated β coefficients and corresponding *P*_adj_ for each mediation step.

For the variant rs1973191819, PM_2.5_ and PM_10_ were suggestively associated with phlegm, FEV_1_/FVC post, cough, and COPD in the mutant-type subpopulation (*P*_adj_mutant_ < 0.1), but not in wild-type individuals (Fig. 5A; Table S11). PM1 showed the strongest negative association with the FEV_1_/FVC post-ratio (β_mutant_ = –2.051), while CO exposure was exclusively positively associated with the risk of COPD (β_mutant_ = 6.554). Increased air pollutant levels were exploratorily associated with the presence of three bacterial taxa (*P. micra*, *F. alocis*, *S. infantis*) in rs1973191819-mutated groups (β_mutant_ > 0, *P*_adj_mutant_ < 0.1). To explore which bacteria may exert a mediation effect, bacterial features were further used as independent variables to examine their associations with respiratory phenotypes. Only the presence of *F. alocis* was significantly associated with both a decline in FEV_1_/FVC post-ratio (β_mutant_ = –5.015, *P*_adj_mutant_ = 0.014, *P*_adj_wild_ > 0.1) and an increased risk of COPD (β_mutant_ = 2.004, *P*_adj_mutant_ = 0.010, *P*_adj_wild_ > 0.1), suggesting a potential mediating role modulated by variant rs1973191819 (Fig. 5A; Table S12). These findings showed a potential genotype-modified effect in which colonization by *F. alocis* mediates the effects of four air pollutants (Cl, O_3_, NO_3_: *P*_adj_mutant_ < 0.05, NO_2_: *P*_adj_mutant_ < 0.1) on impaired lung function (reduced FEV_1_/FVC post-ratio) and elevated COPD risk (*P*_adj_mutant_ < 0.05) (Fig. 5B).

The variant 1:26157175, which is associated with bacterial abundance, has been suggestively associated with 19 traits linking air pollutants (PM_1_, PM_2.5_, PM_10_) to altered lung function (FEV_1_% predicted, FEV_1_/FVC post-ratio, COPD, phlegm) under the genetic interaction model (*P*_adj_mutant_ < 0.1, *P*_adj_wild_ > 0.1) (Fig. 5C; Table S12). The strongest positive association was observed between CO exposure and COPD risk (β_mutant_ = 10.543; Fig. 5C; Table S12). The abundance of three bacterial species increased in response to cumulative air pollution exposure: NO_2_ exposure was nominally associated with a slight increase in *S. sanguinis* (β_mutant_ = 0.003, *P*_raw_mutant_ = 0.025, *P*_adj_mutant_ = 0.062), and occupational exposures were nominally associated with a modest increase in *R. pickettii* (β_mutant_ = 0.004, *P*_raw_mutant_ = 0.015, *P*_raw_mutant_ = 0.062) (Fig. 5C; Table S12). However, only *Haemophilus* exhibited a significant positive association with the PRISm index, where a 10-fold increase in its abundance raised the index by 3.983 units (Fig. 5C). Therefore, no fully bacteria-mediated pathway linking environmental exposures to lung function outcomes was observed in this genotype.

For variant rs1343834070, also associated with bacterial abundance, 13 associations between elevated air pollutants and impaired lung function were identified. Specifically, exposure to PM_2.5_ was linked to multiple respiratory traits, including FEV_1_% predicted, FEV_1_/FVC post-ratio, COPD, and phlegm (Fig. 5D). CO exposure showed the strongest potential effect, with each increment corresponding to a 6.684-unit increase in the cough index (Fig. 5D; Table S12). PM_2.5_ was also nominally associated with a slight increase in *Oribacterium* abundance (β_mutant_ = 0.0003, *P*_raw_mutant_ = 0.01, *P*_adj_mutant_ = 0.071) (Fig. 5D; Table S11). Including *Oribacterium* as an independent variable revealed that a 10-fold increase in its abundance was significantly linked to impaired lung function, corresponding to a 3.775-unit increase in dyspnea (*P*_adj_mutant_ = 0.032) and a 4.384-unit increase in the CAT score (*P*_adj_mutant_ = 0.032) (Fig. 5D; Table S12). A potential pathway, regulated by rs1343834070, is proposed in which *Oribacterium* abundance may mediate the effects of PM_2.5_ on increased dyspnea and elevated CAT score (Fig. 5E).

## DISCUSSION

This metagenome-wide study provides a comprehensive analysis of the associations between environmental exposures, the respiratory microbiome, and respiratory traits, highlighting the modulation of these relationships by host genetic variation. By integrating extensive genomic, metagenomic, environmental exposure, and respiratory phenotype data, hundreds of genome-wide association analyses were conducted, identifying genetic variations associated with environmental exposures, microbiome composition, and respiratory function.

Genetic variants associated with both lung function and air-pollutant exposure were significantly enriched in pathways, such as endocytosis, FcγR-mediated phagocytosis, bacterial invasion of epithelial cells, AMPK signaling, and glycosaminoglycan biosynthesis (heparan sulfate/heparin). These pathways are closely linked to host-microbe crosstalk, suggesting that the relationship between environmental exposures and host genetics may be partially mediated through microbiome-related pathways. Specifically, endocytosis and FcγR-mediated phagocytosis are key processes in microbial recognition, uptake, and clearance by epithelial and immune cells, playing a significant role in the regulation of microbial colonization and inflammation (28, 29). The bacterial invasion of epithelial cells pathway represents host-microbe interactions at the epithelial interface and may contribute to variations in airway microbiome composition and related immune responses (42). Furthermore, glycosaminoglycan biosynthesis, particularly heparan sulfate/heparin metabolism, provides microbial adhesion sites that facilitate colonization and infection initiation (43). Additionally, the AMPK signaling pathway has been implicated in linking microbial-derived metabolites (e.g., lipopolysaccharide, succinate) with host immune regulation (44). These pathways may offer functional links through which host genetic variation and environmental exposures are associated with microbiome-related host responses. Nevertheless, longitudinal cohort studies combined with rigorous experimental validation, such as gnotobiotic animal models or cell-based perturbation experiments, will be essential to confirm these associations and advance from prioritized candidates toward mechanistic understanding.

Using a two-step MR framework, this study provides evidence that several air pollutants (e.g., SO_2_, Cl, CO, and PM_2.5_) impair lung function, with these effects significantly mediated by pathways involving the airway microbiome. In particular, taxa such as *Actinomyces*, *H. influenzae*, *Neisseria perflava*, *F. alocis*, and *Veillonella* spp. were identified as potential mediators linking pollutant exposure to respiratory impairment, consistent with our previous findings (25). *Actinomyces* (e.g., *A. odontolyticus*) are abundant oral commensals frequently detected in induced sputum due to oropharyngeal carryover (45). Consequently, the observed mediation effect involving *Actinomyces* should be interpreted with caution. It may reflect a genuine role in the lower respiratory tract, or it could merely represent shared variation with upper-airway microbiota that respond independently to air pollutants. Additionally, because ambient air pollution is not a classically heritable exposure, SNP-pollutant associations are prone to residual confounding from geographic, occupational, behavioral, and population stratification factors, which cannot be entirely ruled out in the present study. Therefore, the resulting estimates should be considered as instrument-supported associations rather than definitive causal effects. Future investigative efforts incorporating more detailed and granular exposure phenotyping are highly warranted to formally test the robustness of these MR estimates.

Instrumental SNPs associated with these microbial taxa were significantly enriched in the PI3K-Akt signaling pathway and the small GTPase-mediated signaling pathway, both of which are key pathways frequently targeted by pathogens to promote intracellular survival and suppress host immune responses (38–40). These pathways also play roles in the regulation of airway epithelial cell survival, inflammatory responses, and tissue remodeling, processes that are central to the pathophysiology of chronic airway diseases, such as asthma and COPD (36, 37). These findings raise the possibility that genetic variants influencing microbial regulation could be linked to host susceptibility to airway injury and remodeling, possibly through shared signaling mechanisms. Air pollutants, such as SO_x_, CO, and PM_2.5_, are known to impair lung function and increase the risk of respiratory diseases via oxidative stress and inflammatory pathways (1–3), which in turn may disturb the respiratory microbiome balance. However, our MR results suggest that airway microbiome dysbiosis may not be solely a downstream consequence of these processes, but could also be associated with host responses, contributing to respiratory disease (46, 47). These findings support a possible genetic basis for the involvement of airway microbes in the relationship between air pollution exposure and respiratory impairment.

Our findings nominate three loci in *MEOX1*, *FAM110D*, and *USP36* as candidate host-genetic modifiers of airway microbial colonization. First, the variant rs1973191819, located upstream of the *MEOX1* gene, was prioritized as a candidate host-genetic signal potentially related to lower colonization by opportunistic bacteria, such as *F. alocis*, *M. diversum*, *P. stomatis*, and *Eubacterium* spp., suggesting a host genetic influence on microbial colonization dynamics. *MEOX1* encodes a mesodermal homeobox transcription factor involved in extracellular matrix remodeling and fibrogenic activity (48, 49). It has also been reported to promote the persistence of resistant myofibroblasts and contribute to pulmonary fibrosis (50). Moreover, our previous study found that *MEOX1* is significantly associated with lung function in patients with COPD (35). One hypothesis is that genetic variation near this locus could alter *MEOX1* expression and thereby influence epithelial responses that suppress *F. alocis* colonization. The genotype-stratified cohort showed distinct effects of exposures (CO, smoking, Cl, PM_2.5_, etc.) on lung function and COPD (51, 52), with *F. alocis* acting as a potential mediator. Smoking- and PM_2.5_-induced inflammation and oxidative stress are well-established mechanisms contributing to COPD (46, 53, 54). Our findings suggest that host genetic susceptibility to environmental exposures influences microbial dynamics, with individuals carrying the mutant genotype exhibiting reduced colonization of *F. alocis*, indicative of a potential protective association. Nevertheless, exposure to air pollutants may modify this association, as suggested by the observed positive association. Consistent with previous studies showing that *F. alocis* adapts to tobacco smoke and exhibits increased pathogenicity under such exposure (55), these results raise the hypothesis that rs1973191819 marks a genotype-dependent exposure-microbe association during respiratory injury caused by air pollutants and *F. alocis* colonization.

Two loci were identified as being associated with bacterial abundance. The variant at 1:26157175 was associated with increased abundances of *R. pickettii* and *Haemophilus*. This locus maps to *FAM110D*, a member of the *FAM110* gene family, which has been reported to exhibit anticancer-related functions (56). For example, *FAM110B* can inhibit the growth and invasion of non-small cell lung cancer (57), while *FAM110A* has been implicated in the initiation and progression of pancreatic cancer (58). Given the shared involvement of these genes in pathways related to cell proliferation, epithelial integrity, and stress response, *FAM110D* remains a candidate modifier of airway epithelial responses to microbial colonization and environmental stimuli. CO exposure was strongly associated with an increased risk of COPD, consistent with previous studies (52, 59). These data nominate this locus for follow-up on whether this association is modified by genetic variation at the *FAM110D* locus. Although genotype-stratified associations between *R. pickettii* abundance and respiratory traits were not directly observed, individuals with CAT scores greater than 10 exhibited significantly higher abundance of *R. pickettii*. These observations suggest that environmental exposures, such as occupation, NO_2_, smoking, O_3_, SO_2_, and SO_4,_ may act as stressors (59, 60) that may interact with host genetic susceptibility and create conditions compatible with higher abundance of disease-associated bacteria, such as *R. pickettii* (61, 62). Such gene-microbiome-environment associations may be treated as candidate pathways through which environmental stressors and host genetics jointly influence airway inflammation and respiratory disease susceptibility (63–65).

Similarly, another variant, rs1343834070, located downstream of *USP36*, was associated with decreased abundances of *Oribacterium*, *S. infantis*, and *G. sanguinis*, which are rarely detected in individuals with respiratory disease (66), but increased abundances of pathogenic species *R. pickettii and Neisseria*, which are frequently observed in patients with CF or COPD (25, 61, 62). These opposite microbial shifts prioritize rs1343834070 as a candidate susceptibility-associated locus, potentially linking host genetic variation to environmentally associated microbiome dysbiosis (67, 68). Our findings demonstrate that the rs1343834070*-USP36* axis selectively modulates airway microbiota abundance depending on host genetic susceptibility thresholds for different bacterial taxa. Biologically, *USP36* has been implicated in innate immune regulation through nucleolar control and modulation of antimicrobial peptide expression, such as *spp-1* in *Caenorhabditis elegans* and analogous pathways in humans (69, 70). This contrast likely reflects differential host susceptibility thresholds: *R. pickettii*, an opportunistic environmental taxon, is highly sensitive to host mucosal defenses, such that modest changes in *USP36* expression significantly alter its abundance. In contrast, *S. aureus*, an opportunistic pathogen equipped with potent AMP-evasion mechanisms (e.g., the *mprF* system) (71), exhibits a higher susceptibility threshold, rendering its colonization resilient to genetic variation at this locus.

Additionally, *USP36* participates in ubiquitin-mediated protein homeostasis, and dysregulation of this pathway has been implicated in pulmonary diseases, such as COPD and fibrosis (72–74). Therefore, *USP36* is best interpreted here as a prioritized candidate gene whose possible links to tissue remodeling, inflammation, or cellular stress responses (75, 76). The common commensal *Oribacterium* was prioritized as a potential mediator of the associations of PM_2.5_ with dyspnea and CAT score in the mutant-type group, suggesting that PM_2.5_ could be associated with a reduced protective role in the airway microbiome (66, 77), potentially via epigenetic modifications in host epithelial or immune genes. Evidence shows that PM_2.5_ exposure can induce DNA methylation and other epigenetic alterations at gene regulatory regions, including loci involved in immune and epithelial functions (78).

Several limitations should be acknowledged. First, the sample size of this study was relatively small and geographically restricted to Guangdong Province; future research with larger and more diverse populations is necessary to enhance statistical power and generalizability. Second, host genotypes were inferred from metagenomic reads at an average depth of approximately 1.9×, which reduces sensitivity for low-frequency variants and introduces uncertainty at heterozygous sites. Consequently, the fine-mapped loci should be interpreted as prioritized candidates rather than definitive causal variants; confirmation will require dedicated host genotyping at higher sequencing depth. Third, although several loci have been associated with the interplay between environmental exposures, the microbiome, and lung function, our findings remain exploratory. They are designed to highlight promising host-microbe interactions rather than to establish underlying mechanisms. Therefore, transitioning from genetic prioritization to causal mechanisms will necessitate functional follow-up studies, including eQTL co-localization in airway epithelium, host gene perturbation in airway organoid-microbe co-cultures, and genotype-stratified longitudinal sampling. Validation in larger cohorts is also warranted.

In conclusion, our results provide preliminary evidence suggesting a potential genotype-dependent association between environmental exposures, the airway microbiome, and respiratory health. Host genetic variants may interact with microbial responses to environmental stressors, subtly shaping individual susceptibility to lung function decline and increased risk of respiratory diseases. These findings highlight the airway microbiome as a potentially genetically regulated factor linking environmental exposures to respiratory impairment.

## MATERIALS AND METHODS

### Participants and sample collection

The discovery cohort of sputum metagenomes from Guangdong, China, has been described previously (25). Eligible individuals for sputum collection were those with complete questionnaire data, anthropometric measurements, and acceptable spirometry results. The sputum nebulization procedure and quality control measures have been described in prior studies (79, 80). Briefly, nebulization was conducted for 15–20 min using 5% or 3% hypertonic saline based on post-bronchodilator FEV_1_ levels (>65% or ≤65% predicted, respectively). The procedure was completed upon obtaining ≥2 mL of sputum or if discomfort occurred. Selected sputum plugs were processed within 4 h using dithiothreitol and filtered. Samples with a squamous epithelial cell-to-leukocyte ratio  <1:2.5 were considered acceptable. Remaining specimens were stored at −20°C and transferred to −80°C within 4 h. The metagenomic sequencing data from a total of 1,651 quality-controlled sputum samples were re-analyzed.

### Phenotype collection

Microbiome profiling from 16S rRNA gene, ITS amplicon, and metagenomic sequencing data was processed as described in our previous study (25). Bacterial and fungal taxa with an average relative abundance >0.0001 (216 bacterial species from metagenomic data, 68 bacterial genera from 16S rRNA amplicon data, and 232 fungal genera from ITS amplicon data) were included in subsequent analyses. Clinical assessments of participants were conducted at the designated healthcare facility, which included spirometry and sputum induction. Detailed clinical assessment protocols are outlined in our previous study (25). A bronchodilation test with 400 μg salbutamol was conducted using handheld spirometers (MasterScreen Pneumo), unless contraindicated, with post-bronchodilator values assessed after 15 min. At least three acceptable maneuvers were performed, and the highest FEV_1_, FVC, and MMEF values were recorded. Respiratory symptoms (wheezing, cough, phlegm, dyspnea) were assessed using standardized questionnaires. All patients with COPD met the diagnostic criteria according to GOLD (81). The CAT questionnaire was used to evaluate symptom burden, with a score ≥10 indicating high burden (82). Finally, exposure assessment for each participant was collected from questionnaire data. Detailed exposure assessment protocols have been documented in our prior study (25). Briefly, active smoking was defined as current versus non-current smoking; secondhand smoke exposure was determined by cohabitation with daily smokers after age 14. Biofuel exposure referred to household use of biomass or coal for cooking (in the past year) or heating (in winter). Occupational exposure was defined as ≥3 months/year of dust, gas, or fume exposure, with occupation types recorded. Ambient air pollution was assessed as the 2-year average PM_2.5_ concentration at 1 km × 1 km resolution using the China High Air Pollutants data sets (https://weijing-rs.github.io/product.html).

### SNP calling and host genotyping

Metagenomic reads were aligned to the human reference genome (GRCh38) using BWA (v0.7.17) (83) after removing low-quality and adaptor-containing sequences. For reads mapped to the human genome, PCR duplicates were marked using SAMtools (v1.16.1) (84). Variant calling was performed following the standard GATK pipelines (85). Specifically, BaseRecalibrator was used to generate recalibration tables and identify known SNPs in the BAM files using dbSNP (v138) (86). Base Quality Score Recalibration was then applied to adjust base quality scores and remove read pairs with improperly aligned segments. Genetic variants were called using GATK’s HaplotypeCaller, and the resulting GVCFs containing SNPs were further processed through CombineGVCFs and GenotypeGVCFs for joint genotyping. A hard filtering strategy was applied to the raw variants, excluding SNPs with quality metrics below the recommended thresholds (QD < 2.0, FS > 60.0, MQ < 40.0, MQRankSum < –12.5, ReadPosRankSum < –8.0, or SOR > 3.0). After filtration, a total of 195,377,769 variants were retained. Variants were further filtered using Plink (version 2.0.0) (87) based on the following criteria: Hardy-Weinberg equilibrium *P* value > 10^–8^, genotype call rate > 95%, and MAF > 1%. Samples were retained if they had a variant call rate >95% and showed no evidence of population stratification. After quality control, a total of 1,450 individuals and approximately 650,000 variants were retained for subsequent GWAS analyses.

### GWAS of sputum microbial taxa, respiratory traits, and environmental exposure

The arcsine square root function and Z-score normalization were applied uniformly to all continuous relative abundance traits (for both bacterial and fungal taxa) prior to GWAS. To account for key demographic and technical confounders, microbiome traits were then pre-corrected for sex and age using the step3.1_correct_for_covariates.R script from the miQTL_cookbook pipeline (https://github.com/alexa-kur/miQTL_cookbook/). In the subsequent GWAS, we further adjusted for age, sex, BMI, and PCs. These covariates were selected because they are well-established confounders in respiratory microbiome studies that can strongly influence microbial composition. Sensitivity analyses with additional or alternative covariate sets were performed and yielded consistent results. A total of 225 bacterial traits and 66 fungal traits were subjected to GWAS. The bacterial traits included 107 quantitative traits (58 at the species level and 49 at the genus level) and 118 binary traits (109 species-level and 9 genus-level), while the fungal traits comprised 37 quantitative and 29 binary traits, all at the genus level. For lung function, nine phenotypes were subjected to GWAS, including three continuous variables (CAT score, FEV_1_/FVC, FEV_1_ predict) and six binary traits (pre-COPD, preserved ratio impaired spirometry [PRISm], COPD, cough, phlegm, dyspnea). PRISm was defined as an FEV_1_ < 80% predicted in the presence of a preserved FEV_1_/FVC post-ratio (≥0.7) (88). Individuals with preserved lung function but with at least one exposure risk factor and documented respiratory symptoms (cough, dyspnea, phlegm, wheeze, or a CAT score ≥ 10) were classified as being in the “at-risk” stage of pre-COPD (89). For environmental exposure, 15 air pollution-related traits—biofuel exposure, occupational pollution, smoking, secondhand smoke exposure, chemical index (CI), carbon monoxide (CO), ammonium (NH_4_), nitrogen dioxide (NO_2_), nitrate (NO_3_), ozone (O_3_), sulfur dioxide (SO_2_), sulfate (SO_4_), and particulate matter (PM_1_, PM_2.5_, PM_10_)—were included in the genome-wide association analysis. The quantitative traits for genome-wide association analyses were conducted using the fastGWA algorithm within the GCTA (v1.94.1) software framework (90), while the binary traits for GWAS were analyzed using Plink (v2.0.0) (87).

### Heritability estimates and independent tests for microbial taxa

The abundance-transformed microbial features were estimated for heritability using the REML method implemented in GCTA (91, 92). REML is a statistical approach that partitions the total phenotypic variance into genetic and residual components by fitting a linear mixed model. This method utilizes genome-wide SNP data to construct a genetic relationship matrix among individuals, enabling the estimation of the proportion of phenotypic variance explained by all genotyped common variants collectively. The phenotype y was modeled as


y=Xβ+g+ε,

where *X*β represents fixed effects, $g∼N(0,G\sigma_{g}^{2})$ is the genetic effect, with *G* being the genetic relationship matrix, and $\varepsilon ∼N(0,I\sigma_{e}^{2})$ is the residual error. Heritability (*h*^2^) is calculated as the proportion of variance explained by genetic effects:


$$h^{2}=\sigma_{g}^{2}/(\sigma_{g}^{2}+\sigma_{e}^{2}).$$

REML provides unbiased estimates of genetic and residual variance components by maximizing the likelihood of the observed data, adjusting for fixed effects. A likelihood ratio test (LRT) was performed to test whether the heritability of a given phenotype was significant (*P*_LRT_ < 0.05).

To assess potential systematic bias in the genome-wide association analysis, the genomic inflation factor (λ) was calculated (93). λ was defined as the ratio of the median of the observed chi-square (X^2^) test statistics to the expected median under the null hypothesis. For association tests with one degree of freedom, the expected median of the chi-square distribution is 0.455. *P* values from the GWAS were first converted to chi-square statistics using the formula:


$$\chi^{2}=qchisq(1-P,\;df=1).$$

The genomic inflation factor was then computed as


$$\lambda =\frac{\mathrm{median}(\chi_{observed}^{2})}{0.455}.$$

The genomic inflation factor (λ) provides a quantitative measure of test statistic inflation, with values near 1 indicating minimal confounding and supporting the validity of GWAS results.

### Meta-analysis of microbiome GWAS

Genome-wide meta-analyses of microbiome traits were performed using METAL (v 2011.03.25) (94), employing an IVW fixed-effect model based on standard errors. Two meta-analyses were conducted using 291 microbial GWAS summary results, comprising 225 bacterial traits (107 quantitative traits and 118 binary traits) and 66 fungal traits (37 quantitative traits, 29 binary traits). Prior to meta-analysis, summary statistics were harmonized for genomic build, allele orientation, and variant identifiers. Variants with ambiguous alleles or low MAF were excluded. Based on the number of independent tests, Bonferroni-adjusted study-wide significance thresholds for bacterial and fungal traits were set to *P*_bac_ < 5 × 10^–8^/225 = 2.2 × 10^–10^ and *P*_fun_ < 5 × 10^–8^/66 = 7.6 × 10^–10^, respectively. The identified SNPs were clustered into genetic loci within 200-kilobase windows based on linkage disequilibrium (LD > 0.2) with the lead SNPs. Target SNPs were defined as genomic signals showing genome-wide significant associations (*P* < 5 × 10^–8^) with at least two taxonomically distinct microbial traits at either the species or genus level. In total, 87 target SNPs related to bacterial traits and 258 target SNPs related to fungal traits were identified.

### Two-step one-sample Mendelian randomization

A two-step, one-sample MR framework (95) was employed to investigate the potential pathway from air pollution to lung function, mediated by microbial traits. Microbial GWAS summary statistics (216 bacterial species, 68 bacterial genera, and 232 fungal genera) were used as mediators in subsequent analyses. Summary statistics for 9 lung function phenotypes were used as outcomes, and those for 15 air pollution traits were considered as exposures. Genetic instruments were selected based on genome-wide significant variants (*P* < 1 × 10^–5^), followed by LD pruning to ensure independence among variants. The MR analyses were performed using the R package MendelianRandomization (96). In the first step, the association between air pollution exposures and microbial mediators was estimated using the IVW method (97). Given that air pollution is not a classical heritable exposure, these analyses should be interpreted as instrument-supported associations rather than strict causal effects. The second step evaluated the effect of microbial traits on lung function outcomes using the IVW method. Multiple-testing correction was performed across all tested exposure-outcomes associations using the Benjamini-Hochberg false discovery rate (FDR) method, with statistical significance defined as *P*_adj_ < 0.05. The mediated (indirect) effect in MR, denoted as β*_m_*, was calculated as the product of effect estimates (β coefficients) from the two steps. Instrumental variants associated with microbial traits that showed mediation effects were annotated using SnpEff (v17.0.1) (98) based on the GRCh38.86 reference genome, assigning variants to genes and predicting their functional consequences. Functional enrichment of the resulting gene set was conducted using clusterProfiler (v4.3.3) (99), with gene ID mapping via the org.Hs.eg.db database. Pathways and GO term enrichment were evaluated, with significance defined as adjusted *P* < 0.05.

### Fine-mapping and genotype analysis

To identify putative variants within microbiota-associated loci, fine-mapping was conducted using FINEMAP (v1.4.2) (100), a Bayesian statistical tool designed for variable selection in high-dimensional genetic data. The --config was used to specify the configuration file containing input parameters and data paths, ensuring reproducibility of the analysis. The --rsids option restricted the analysis to lead variants, enabling variant-level fine-mapping within each locus. Regional Manhattan plots were generated using LDblockshow (v1.4) (https://github.com/BGI-shenzhen/LDBlockShow) to visualize association signals and local LD structures (101). For each lead SNP, variants within a 200 kb flanking region were included, and LD was calculated using genotype data from ancestry-matched individuals. The plots display –log_10_(*P* values) of SNPs, color-coded by LD (r^2^) with the lead variant, aiding in the interpretation of the fine-mapping results. The genotype distribution of putative variants identified through fine-mapping was extracted from the study cohort to characterize allele frequencies and carrier status.

### Genotype-stratified association analysis among environmental exposures, microbial taxa, and respiratory traits

Putative variants identified through fine-mapping were selected for further analysis. The genotype distributions of these variants in the study population were extracted, and individuals were stratified into wild-type and mutation-type carrier groups based on the presence or absence of the variant allele. Within each genotype-defined subgroup, GLMs were employed to assess the associations among 15 environmental exposures, 9 lung function phenotypes, and microbial traits previously linked to the corresponding loci (102). GLMs were fitted with appropriate distribution families (e.g., Gaussian for continuous traits and binomial for binary traits), adjusting for relevant covariates, including age, sex, and BMI, to account for population stratification (103).

Three GLMs were constructed within genotype-defined groups to explore potential pathways: (i) air pollutant levels as independent variables and lung function traits as dependent variables; (ii) air pollutant levels as independent variables and microbial abundances as dependent variables; (iii) microbial abundances or binary traits as independent variables and lung function traits as dependent variables. This study focused on associations between environmental exposures and respiratory traits or microbial taxa, as well as between microbial taxa and respiratory traits, that were significant in the variant group but non-significant in the wild-type group. Multiple testing correction was performed using the Benjamini-Hochberg FDR method. A microbe-mediated association between air pollutants and respiratory traits was identified as potential when both the environmental exposure-microbial taxa and the corresponding microbial taxa-respiratory trait associations were suggestive significant in the variant group (*P*_adj_mutant_ < 0.1) but not in the wild-type group (*P*_adj_wild_ > 0.1).

### Public data validation

To validate the findings from the microbiome genome-wide association analyses, multiple publicly available data sets were utilized (PRJCA007879, PRJNA714488, PRJNA858501, PRJNA991321, CNP0001664, and EGAS00001006398) (Table S13). Microbial taxa identified in our study were compared with those from the reported data sets, and only taxa shared across data sets were retained for replication. For each overlapping taxon, genome-wide significant variants (*P* < 1 × 10^−4^) were independently extracted from both the discovery and replication cohorts. Variants reaching the significance threshold in both cohorts were annotated using SnpEff based on the GRCh38.86 reference genome. SNP- and gene-level annotations were compared across data sets, and matching microbiome-SNP and microbiome-gene pairs were retained and counted. Functional enrichment analysis of the overlapping gene set was performed using the R package ClusterProfiler with the org.Hs.eg.db database. Enrichment of biological pathways and GO terms was assessed, with significance defined as an adjusted *P* value < 0.05.

## Data Availability

The raw metagenomic data have been deposited in the Genome Sequence Archive at the National Genomics Data Center (https://ngdc.cncb.ac.cn/), Beijing Institute of Genomics (BIG), Chinese Academy of Sciences, under accession PRJCA012829. GWAS summary statistics have been deposited in Figshare (https://doi.org/10.6084/m9.figshare.30892412, https://doi.org/10.6084/m9.figshare.30892331). The analysis pipeline and scripts used in this study are available on GitHub (https://github.com/vincenthowudoin-beep/CDC-microbiome-GWAS).

## SUPPLEMENTAL MATERIAL

Supplemental Figures msystems.00442-26-s0001.docx Figures S1-S6.

Supplemental Tables msystems.00442-26-s0002.xlsx Tables S1-S13.

ASM does not own the copyrights to Supplemental Material that may be linked to, or accessed through, an article. The authors have granted ASM a non-exclusive, world-wide license to publish the Supplemental Material files. Please contact the corresponding author directly for reuse.
