# Supplementary material for: The associations of human genetic variations with airway microbiome, environmental exposures, and respiratory health
Source: mSystems. 2026 Jul 29;11(8):e00442-26. doi: 10.1128/msystems.00442-26 (PMC13483325; doi:10.1128/msystems.00442-26)
Supplement: Supplementary file 1 [file msystems.00442-26-s0001.docx]

**The associations of human genetic variations with airway microbiome, environmental exposures, and respiratory health**

**Supplemental Information**

Shifen Xu^1^*, Leiyang1*, Jingyuan Gao^1^*, Yunfeng Shi^1^*, Xin Tang^1^*, Hanqin Cai^1^, Liling Yang^1^, Yuanyuan Han^1^, Lifeng Lin^2^, Ruilin Meng^2^, Jiufeng Sun^2^, Wei-jie Guan^3,4^, Tao Tang^5^,

Wensheng Shu^1^, Chao Cao^6#^, Xue-yan Zheng^2#^, Zhang Wang^1#^, Xinzhu Yi^1#^

**Figure S1. Overview of genotype calling, quality control, and population structure analysis based on sputum metagenomic SNP data**

**A.** Workflow outlining genotype calling steps, applied software, and quality-control parameters, with the number of SNPs retained after each stage.

**B.** Density plot showing the distribution of SNPs across chromosomes, PCA of 1,450 samples, and embedded scree plot of variance explained by the top ten eigenvalues. The circular plot displays the distribution of SNPs across functional genomic regions.

**C.** Upper bar plot showing SNP missing rate per sample and lower composition plot showing SNP genotype distributions ordered by heterozygosity proportion.

**D.** Boxplots of minor allele frequencies (MAF) across chromosomes, histogram of Hardy–Weinberg equilibrium (HWE) p-values, and density plot of observed minus expected heterozygosity.

**Figure S2. KEGG and GO pathway enrichment analyses of GWAS-mapped genes for air pollutant and lung phenotype–associated loci, and shared microbial taxa across independent cohorts.**

**A–B**. KEGG pathway enrichment of GWAS-mapped genes (p < 10^–4^) for air pollutant and lung phenotype traits.

**C–E**. KEGG and GO enrichment of overlapping genes mapped from shared microbial taxa among validation cohorts and our study (PRJCA012829).

**F.** Bubble plots showing overlapping bacterium–SNP pairs between cohorts, with –log_10_ *p*-values on the axes, bubble size proportional to summed –log_10_ *p*-values, and bubble color indicating taxa identity. Mean relative abundance, effect sizes, and p-values are indicated at the top.

**Figure S3. Overlap of GWAS-annotated genes** **from shared microbial taxa across independent cohorts**

The overlapped genes from shared microbial taxa between our study and the EAS combined cohort **(A)**, CNP0001664 cohort **(B)**, and EGAS00001006398 cohort **(C)**.

Bars represent shared microbial taxa ordered by the number of overlapping genes, with the top five highlighted in red. The lower right bar chart summarizes the total number of significant loci and genes per cohort pair; the upper pie chart shows the distribution of cohort-unique and shared variant loci. Mean relative abundance, effect sizes, and *p*-values are indicated at the top.

**Figure S4. Potential microbial-mediation effects of association between exposures and health outcomes and pathway enrichment of associated fungal taxa**

**A, C.** Sankey diagram illustrating mediation effects of bacterial and fungal taxa on health outcomes. The left, middle, and right panels represent exposures, bacterial taxa, and pulmonary function indices, respectively. The color gradient encodes the magnitude of the mediation effect (β_m_).

**B.** Bar plot showing the number of fungus-mediated Mendelian Randomization (MR) associations between air pollutant exposures and health outcomes. Different colors correspond to distinct health outcomes.

**D.** Bubble plot showing KEGG and GO pathway enrichment analyses of genes mapped from genome-wide significant loci (*p* < 1 × 10^–5^) associated with fungus taxa exhibiting mediating effects.

**Figure S5. Regional association plots of the significant bacterial species and genus with the three causal loci and genotype-stratified exposure–health outcome analyses**

**A–C.** Regional association plots of significant bacterial taxa with loci rs1973191819, 1:26157175, and rs1343834070. The red dashed line marks the GWAS significance threshold (*p* < 5 × 10^–8^), and point color represents LD strength with the causal variant. Bar and box plots show microbial prevalence or relative abundance across genotypes, with p-values from Fisher’s exact and Wilcoxon rank-sum tests.

**D.** Sensitivity analysis of the associations between the lead SNPs (rs1310134471 and rs1343834070) and three bacterial taxa, using several different covariate models. The main model (used in this study) included age, sex, BMI, and PCs; model 1 included only age and sex; model 2 included age, sex, and BMI; and model 3 included no covariates.

**Figure S6. Association of rs1343834070 with bacterial abundance and *USP36* cis-eQTL evidence.**
**A.** GWAS association strength of the lead SNP rs1343834070 for *Ralstonia pickettii* and *Staphylococcus aureus*. The y-axis shows −log10(*P*_GWAS_). rs1343834070 was strongly associated with *Ralstonia pickettii* abundance (*P* = 3.92×10^–16^), but not with *Staphylococcus aureus* (*P* = 0.883).
**B.** cis-eQTL evidence for *USP36* across four eQTL datasets: GTEx Blood, GTEx Lung, PEER50, and HCP100. The y-axis shows −log10(*P*_eQTL_). *USP36* showed strong cis-eQTL signals in GTEx Lung, PEER50, and HCP100, whereas no *USP36* signal was available in GTEx Blood. Blue and red points indicate the corresponding bacterial GWAS contexts for *Ralstonia pickettii* and *Staphylococcus aureus*, respectively.

**Supplementary figure 1**

**Supplementary figure 2**

**Supplementary figure 3**

**Supplementary figure 4**

**Supplementary figure 5**

**Supplementary figure 6**
